# Radiation therapy and chemotherapy-induced oral mucositis

**DOI:** 10.1016/S1808-8694(15)30110-5

**Published:** 2015-10-19

**Authors:** Luiz Evaristo Ricci Volpato, Thiago Cruvinel Silva, Thaís Marchini Oliveira, Vivien Thiemy Sakai, Maria Aparecida Andrade Moreira Machado

**Affiliations:** a Master’s degree, doctoral student in odontopediatrics, Department of Odontopediatrics, Orthodontics and Collective Health at the Bauru Dental School, Sao Paulo University. Professor of odontopediatrics and dentistry for special patients, Cuiaba University.; b Dental surgeon, Master’s degree student in odontopediatrics, Department of Odontopediatrics, Orthodontics and Collective Health at the Bauru Dental School, Sao Paulo University.; c Master’s degree, doctoral student in odontopediatrics, Department of Odontopediatrics, Orthodontics and Collective Health at the Bauru Dental School, Sao Paulo University.; d Master’s degree, doctoral student in odontopediatrics, Department of Odontopediatrics, Orthodontics and Collective Health at the Bauru Dental School, Sao Paulo University.; e Doctor, associate professor in the Department of Odontopediatrics, Orthodontics and Collective Health at the Bauru Dental School, Sao Paulo University. Bauru Dental School - Sao Paulo University.

**Keywords:** adverse effects, mucositis, chemotherapy, radiotherapy

## Abstract

TIncreasing the intensity of radiation therapy and chemotherapy in the management of cancer has increased the incidence of adverse effects, especially oral mucositis.

**Aim and methods:**

a bibliographical review was conducted on the definition of oral mucositis, its clinical findings, the incidence, its etiology, the pathofisiology, associated morbidity, prevention and treatment.

**Results:**

current studies define oral mucositis as a very frequent and painful inflammation with ulcers on the oral mucosa that are covered by a pseudo membrane. The incidence and severity of lesions are influenced by patient and treatment variables. Oral mucositis is a result of two major mechanisms: direct toxicity on the mucosa and myelosuppression due to the treatment. Its pathofisiology is composed of four interdependent phases: an initial inflammatory/vascular phase; an epithelial phase; an ulcerative/bacteriological phase; and a healing phase. It is considered a potential source of life-threatening infection and often is a dose-limiting factor in anticancer therapy. Some interventions have been shown to be potentially effective to prevent and treat oral mucositis. Further intensive research through well-structured clinical trials to obtain the best scientific evidence over the standard therapy of oral mucositis is necessary to attain ideal parameters for radiotherapy and chemotherapy.

## INTRODUCTION

Estimates show that there will be 472,050 new cases of cancer in Brazil in 2006.^1^ Added to the morbidity of cancer, its treatment causes significant side effects, many of which present in the mouth. In other words, mouth cancer causes alterations in the oral cavity, but the treatment used for neoplasms in general may also yield similar effects.^2^, ^3^

There are three commonly used approaches to treating malignant neoplasms: surgery, radiotherapy and chemotherapy. Surgical treatment of cancer has two main objectives: to resect the tumor and other tissues involved, such as lymph nodes, and to remove endocrine organs that may alter and disseminate the disease. Different from surgery, radiotherapy and chemotherapy are not tissue-specific; they act by inhibiting rapidly-dividing cell growth, interfering with cell division mechanisms.^4^ As chemotherapy and radiotherapy do not differentiate between rapidly dividing cancer cells and normal cells that divide at higher rates, such as those in the mouth or the bone marrow, they may result in side effects that present in the mouth.^2^, ^3^, ^4^, ^5^, ^6^, ^7^, ^8^ Furthermore, the mouth harbors many bacteria, which may facilitate access to infectious microorganisms into a myelosuppressed host.^3^, ^4^, ^9^

According to Sonis et al.^4^, the frequency of mouth problems in patients undergoing chemotherapy depends on a number of variables. These variables may be related to the patient or to the treatment. Patient-related factors are age, the diagnosis and the state of the patient’s mouth before and during treatment. Treatment-related variables are the type of drug, the dose and frequency of therapy, and the use of concomitant treatment.

Commonly used medication in chemotherapy includes antimetabolic drugs, such as methotrexate, which inhibit DNA synthesis and tend to produce mucositis. Similar effects are seen with alkylating agents, such as 5-fluorouracil, used frequently in the treatment of gastrointestinal tract malignant tumors. Antibiotics, such as adriamycin, may cause direct effects in the mouth, or side effects as a result of its action on accessory salivary glands. Plant alkaloids rarely cause direct derangement of the oral mucosa, but may affect the mouth as a result of their neurotoxic effects.^4^

A common practice is to increase the intensity of therapy to raise primary cure rates.10 Unfortunately, the toxicity of chemotherapy tends to yield severe adverse effects, which limits medical treatment. In these cases, the most effective dose cannot be given, which reduces the response of tumors to therapy.^3^, ^8^, ^10^, ^11^, ^12^, ^13^, ^14^

The most common oral complications associated with cancer therapy are as follows: stomatitis, infection, bleeding, mucositis, pain, loss of function and xerostomy.^15^ Sonis et al.^4^ divided oral complications of cancer chemotherapy into two main forms according to the origin, as follows: problems resulting from the direct action of the drug over mouth tissues - direct stomatotoxicity - and mouth problems caused by changes in other tissues - indirect stomatotoxicity - such as the bone marrow. The most common form of direct stomatotoxicity is mucositis, a diffuse ulcerative condition usually in the non-keratinized oral mucosa, involving mostly the soft palate, the jugal mucosa,^4^, ^9^, ^16^, ^17^ the lateral border of the tongue, the wall of the pharynx, the tosillar pillars, 4 the lips, the middle portion of the tongue and the floor of the mouth.^9^, ^16^, ^17^

This paper aims to present physicians and other professionals who support the treatment of cancer patients with information about chemotherapy and radiotherapy-induced oral mucositis. This review of the pertinent literature includes the definition, clinical findings, incidence, etiology, pathophysiology, associated morbidity, prevention and treatment of this form of oral mucositis.

## REVIEW OF THE LITERATURE

### Definition and clinical features

Oral mucositis is defined as inflammation and ulceration of the mouth mucosa^12^, ^15^ with pseudomembrane formation;^11^ it is a potential source of infection which may lead to death.^12^, ^15^ This condition is a frequent and painful debilitating effect of radiotherapy and chemotherapy for cancer, affecting over 40% of patients.^12^

The initial presentation is erythema followed by white desquamating plaques, which are painful when touched. Epithelial crusting and a fibrin exsudate result in a pseudomembrane and ulceration, which is the more pronounced form of mucositis. Patients invariably complain of pain. Exposure of the richly innervated underlying stromal connective tissue due to loss of epithelial cells is found in the most severe form of mucositis;^16^ this condition is usually seen 5 to 7 days following medication.^9^

Ulcers lead to severe pain that frequently requires dietary changes and parenteral narcotics for relief. Ulcerated mucositis in myelosuppressed patients may become a portal for systemic invasion by bacteria or bacterial cell wall products.^11^

According to Bensadoun et al.^17^ the pathology of mucositis shows a shallow ulcer, probably caused by depletion of the basal epithelial layer, with subsequent denudation. The healing response to this injury comprises an inflammatory cell infiltrate, an interstitial exsudate and the remains of cells and fibrin, which result in a pseudomembrane that is analogous to an eschar in a superficial skin lesion. This membrane appears white or opaque when hydrated by saliva. It may appear yellowish or greenish due to superficial infection, particularly if there are deep ulcers.^18^ Food, beverages or topical medication may also change the color. Careful inspection frequently reveals the slightly elevated membrane above the level of the underlying mucosa if the lesion is ulcerated. It may easily be mistaken for candidiasis. Secondary infection of the ulcer by Candida albicans may occur.^11^ Mild trauma may remove the membrane, causing bleeding and ulceration. Increased ulcer size results in a wider adjacent pseudomembrane that covers neighboring geographically connected ulcers. Insufficient cell regeneration results in epithelial atrophy and thinning of the mucosa.^16^

The reaction is confined to the irradiated area in radiotherapy, frequently seen as a mucosal margin of intense erythema.^16^ The severity depends on a variety of factors, including the dose of medication, dose interval, the volume of treated tissue and the type of radiation.^16^, ^19^ Other factors are prior exposure to chemotherapy, concomitant chemotherapy, and systemic diseases such as diabetes mellitus or vascular conditions.^19^ In most patients healing occurs two or three weeks after the end of conventionally fractionated radiotherapy.^16^

Various methods to assess the grade of mucositis in cancer patients have been described; these are based on the presence of signs such as erythema and lesions in isolation or associated with symptoms such as pain and swallowing difficulties.^16^, ^20^

Oral mucositis may be classified into five grades according to the World Health Organization grading system, as follows: grade 0 - indicated absence of mucositis; grade

I - presence of a painless ulcer, erythema or mild sensitivity; grade

II - presence of painful erythema or ulcers that do not interfere with the patient’s ability to take food; grade

III - confluent ulceration that interfere with the patient’s ability to take solid food; and grade IV - severe symptoms requiring enteral or parenteral support.^17^

### Incidence

Anticancer treatment-induced oral mucositis is a frequent complication;15 its incidence varies with patient-related and treatment-related variables (Table 1).

**Table 1:** Variables that affect the incidence of anticancer treatment-induced oral mucositis.

Patient-related variables	Treatment-related variables
Diagnosis^4^	Chemotherapy drug^4^
Age^4^	Drug dosage and frequency^4^
Level of oral health^4^, ^11^	Irradiated region^17^, ^22^
Systemic conditions^15^	Irradiated tissue volume^17^, ^22^
	Type of ionizing radiation^17^, ^22^
	Fractionated dose and cumulative dose of radiotherapy^17^, ^19^, ^22^
	Concomitant chemo and radiotherapy^23^

Generally, malignancies of the blood - such as leukemia and lymphoma - that suppress the bone marrow, tend to show a high rate of oral complications.4 These patients develop problems in the mouth two or three times more frequently than patients that have solid tumors.^21^ This is probably associated with the disease, drug-induced immunosuppression and the frequent use of chemotherapy that specifically targets the cell cycle.^22^

According to Childers et al.^2^ and Sonis et al.^4^, younger age increases the possibility of chemotherapy affecting the mouth. Around 40% of all chemotherapy patients present side effect in the mouth; this number reaches 90% in children under 12 years of age. It seems likely that the high mitotic rate of oral mucosa cells in this age group is an adjuvant factor.4 Notwithstanding its high prevalence, a few studies reporting a lower incidence and severity of oral mucositis in children have been published.^23^

As to the type of treatment, Adamietz et al.^15^ have reported that mucositis may be seen in nearly every patient when chemotherapy and radiotherapy are used simultaneously.

Parulekar et al.^16^ have estimated that chemotherapy-induced mucositis varies from 40 to 76% in patients treated respectively with standard and high-dose chemotherapy.

Nearly all (90% to 97%9,24) patients receiving radiotherapy in the head and neck will develop some degree of mucositis.^16^ Of these patients treated with radiotherapy with or without chemotherapy, 34% to 43% will present severe mucositis. As a result, the patient’s quality of life is affected, hospital admittance rates are higher, the use of total parenteral nutrition is increased and interruption of treatment is more frequent, all of which compromise tumor control. Mucositis causes 9% to 19% of chemotherapy and radiotherapy interruption.^24^

### Etiology

Mucositis is a consequence of two major mechanisms: direct toxicity due to the treatment and myelosuppression that results from therapy. Its pathogenesis is reduced cell renewal in the basal epithelial layers as a result of chemotherapy and radiotherapy; adequate replacement of desquamated cells becomes insufficient.^4^

Cell kinetics initially defines the relative sensitivity of normal tissues to anticancer therapy. Oropharyngeal, intestinal and bone marrow cells divide rapidly and are more sensitive to radiation and chemotherapy compared to cells that divide slower and senescent cells in other parts of the body. Both radiation and chemotherapy cause cell death by interfering on their growth and differentiation mechanisms. Dividing cells are more sensitive to the effects of anticancer therapy.^16^

Cells in the oral mucosa have high mitotic, cell renewal and epithelial maturation rates. This mucosa is, thus, vulnerable to the side effects of chemotherapy.^5^, ^20^ The non-specific effects of cytotoxic drugs used in therapy reduces the rate of epithelial cell renewal, leading to muscle atrophy, localized or diffuse mucosal ulcers and inflammation.^25^ Chemotherapy not only alters mucosal integrity, but also changes the microbial flora in the mouth; it also alters the quantity and composition of saliva and epithelial maturation.^20^ The mucosal barrier is compromised, which is also a risk factor for morbidity and mortality in cancer and myelosuppressed patients.

According to Adamietz et al.^15^ the origin of cancer radiotherapy-induced mucosal lesions is radiobiological or toxic, although infection may further development. Damage to the oral mucosa may lead to infection, which is maintained by immune unbalances, in particular leukopenia. Damage to salivary glands reduce the flow and composition of saliva and its pH, which is followed by changes in the oral microflora, again increasing the risk of infection. Chemotherapy-induced damage differs from that of radiation in that radiation damage is considered as permanent risk areas throughout the patient’s life.^25^

### Pathophysiology

In 1998 a new theoretical model was described for the pathophysiology of radiotherapy-induced mucositis. This model included the knowledge at the time about cytokine activity and integrated local immunological and microbacterial effects with the theory of direct and indirect toxicity. Thus, the pathophysiology of oral mucositis was described as having four interdependent phases, as follows:^11^

1. a vascular/inflammatory phase,
2. an epithelial phase,
3. an ulcerative/bacteriological phase,
4. and a repair phase.

Oral mucositis appears to be the consequence of a series of biological events that begin in the submucosa and progress towards the epithelium.^26^ During such events, radiotherapy activates transcription factors.

During the first phase, pro-inflammatory cytokines, including interleukin 1 (IL-1) and the tumor necrosis factor a (TNF-a), are produced and mediate a local response to therapy, followed by apoptosis and cell damage. The dynamic biological cascade results in injury to the mucosa, frequently presenting as ulcers. A fibrin exsudate containing bacteria - also called the pseudomembrane - covers the ulcer. Cell wall remains originating from colonizing bacteria penetrate the submucosa, where they activate macrophages in the infiltrate; these macrophages produce additional cytokines. These and other related cytokines are responsible for causing direct tissue damage, dilation of blood vessels and other inflammatory effects, all of which increase the deposition of cytotoxic drugs on the mucosa. These effects mediate the transition to the next phase. Some authors have suggested that genetic differences in the inflammatory response may be responsible to variations in susceptibility to chemotherapy-induced oral mucositis.^11^

The second phase (epithelial phase) is related to the previous model of direct stomatotoxicity in which cytotoxic agents inhibit the replication of epithelial basal cells, leading to a reduced capability for tissue renewal.

It is assumed that the third phase (ulcer with infection) derives from trauma on the epithelium, which at this point is thinner and atrophic due to chemotherapy. It is further postulated that bacterial colonization of ulcerated surfaces increases the quantity of pro-inflammatory cytokines in the mucosa, which facilitates systemic infection in a myelosuppressed host.

The repair phase takes place after 2 to 3 weeks in non-myelosuppressed patients that receive no further therapy.^22^ Secondary infection may delay healing of mucosal lesions. Larger and deeper ulcers usually require more time to heal. The profound depletion of mesenchymal cells may result in second intention healing. Depending on the extent of injury, the resulting mucosa may appear pallid, atrophic and less complacent. A few deep ulcers may never heal and may progress to soft tissue or bone necrosis. Although systematic clinical trials of these pathological changes have not been conducted in human beings, deep ulcers that require prolonged healing times probably produce what is clinically noted as late effects on the mucosa. The peak of severity of lesions only indicates partially the degree of mesenchymal cell depletion. The duration of injury also reflects the degree of cell depletion and the recovery rate.^17^

Although there have been recent conceptual developments, certain pathophysiological processes that favor anticancer therapy-induced mucositis have not been fully clarified.^6^, ^24^, ^27^, ^28^, ^29^, ^30^ According to Barasch and Peterson^27^ clinical controversy persist. Cell and molecular studies are difficult to undertake in cancer patients. Furthermore, animal models are inherently limited. Thus, additional evidence from epidemiology and risk factors are needed to further understand the pathophysiology of mucositis.

### Morbidity associated with mucositis

Mouth lesions may become the entry point for the dissemination of bacterial, fungal and virotic infection,^2^, ^8^, ^30^, ^31^ particularly in patients undergoing myelosuppressive or immunosuppressive chemotherapy regimens for the treatment of cancer. In many medical centers, cancer patients undergoing high-dose chemotherapy usually receive antibiotics and antifungal medication preventively or during the periods of drug-induced leukopenia.^21^ A study by Nicolatou-Galitis^30^ revealed that 29.1% of patients presented herpes simplex virus infection, which worsens radiotherapy-induced ulcerative oral mucositis.

This significant acute clinical side effect of anticancer therapy leads to discomfort and pain and hinders adequate nutrition; it may even cause an interruption of mediation or changes in the drug regimen. These effects may increase hospital stay and increase considerably the risk of infection and death in some patients.4 Furthermore, secondary infection may result in severe systemic infection.^2^, ^3^, ^7^, ^8^, ^10^, ^14^, ^16^, ^17^, ^19^, ^30^, ^31^, ^32^, ^33^, ^34^ Mucositis may lead to potentially fatal bacteremia in myelosuppressed patients. All of these conditions may obstruct further treatment or increase its cost, threatening the survival of patients or permanently changing their quality of life and that of their families.

It is clinically difficult, if not impossible, to discriminate between infected and non-infected mucositis. An association between these two types occurs almost always, resulting in a thinned mucosa, pseudomembranes, erythema and ulcers.

### Prevention and Treatment

Although this condition has been studied in detail, no approach has been demonstrated to be effective in the prevention and/or treatment of oral mucositis. Recent systematic reviews and clinical trials have identified potentially effective approaches for the prevention of mucositis, namely: the use of allopurinol, growth factors and povidine-based mouthwashes, hydrolytic enzymes, amiphostine,^15^, ^29^, ^36^ sucralfate^13^, ^37^ and antibiotics,^29^, ^36^, ^38^, ^39^ honey,^19^ low-intensity laser,^17^, ^22^, ^29^, ^35^, ^36^, ^38^, ^40^, ^41^ oral hygiene,^25^, ^42^ analgesics^10^ and anti-inflammatory drugs, and other options.^5^, ^8^, ^11^, ^15^, ^20^, ^28^, ^29^ The evidence for these measures, however, is variable and the benefits for patients with different cancers is unknown.1^3^, ^14^, ^27^, ^36^, ^40^, ^43^, ^44^ The prevention and treatment of mucositis remains an unsolved problem that limits the efficacy of anticancer therapy and reduces the quality of life of these patients.^12^

One of the major concerns during the Mucositis Committee meetings in 2000^45^ was the lack of trials and the empiricism of the methods used at the time for the prevention and treatment of mucositis; many of the strategies were considered as reasonable (such as mouthwashes with saline). It was underlined, however, that a strict review would probably reject those measures. The authors underlined the challenge to reach an adequate protocol that researchers would accept and put in practice.

## FINAL COMMENTS

Oral mucositis is a frequent and debilitating intercurrence in patients undergoing chemotherapy and radiotherapy for the treatment of various forms of cancer. With the use of more aggressive approaches, mucositis as a limiting factor is becoming more evident; its control is one of the priorities in clinical oncology. The past decades have seen advances in the knowledge of the pathophysiology of this condition. There is, however, no consensus on the effectiveness of a variety of measures used in the prevention and treatment of oral mucositis. New well-structured trials are needed to define the standard treatment for managing mucositis. This will alleviate pain, favor nutrition and increase the quality of life of these patients.
